# Chronic Venous Disease during Pregnancy Causes a Systematic Increase in Maternal and Fetal Proinflammatory Markers

**DOI:** 10.3390/ijms23168976

**Published:** 2022-08-11

**Authors:** Miguel A. Ortega, Ana M. Gómez-Lahoz, Lara Sánchez-Trujillo, Oscar Fraile-Martinez, Cielo García-Montero, Luis G. Guijarro, Coral Bravo, Juan A. De Leon-Luis, Jose V. Saz, Julia Bujan, Natalio García-Honduvilla, Jorge Monserrat, Melchor Alvarez-Mon

**Affiliations:** 1 Department of Medicine and Medical Specialities, Faculty of Medicine and Health Sciences, University of Alcalá, 28801 Alcalá de Henares, Spain; 2 Ramón y Cajal Institute of Sanitary Research (IRYCIS), 28034 Madrid, Spain; 3 Service of Pediatric, Hospital Universitario Principe de Asturias, 28801 Alcalá de Henares, Spain; 4 Department of Systems Biology, Faculty of Medicine and Health Sciences (Networking Research Center on for Liver and Digestive Diseases (CIBEREHD)), University of Alcalá, 28801 Alcalá de Henares, Spain; 5 Department of Public and Maternal and Child Health, School of Medicine, Complutense University of Madrid, 28040 Madrid, Spain; 6 Department of Obstetrics and Gynecology, University Hospital Gregorio Marañón, 28009 Madrid, Spain; 7 Health Research Institute Gregorio Marañón, 28009 Madrid, Spain; 8 Department of Biomedicine and Biotechnology, Faculty of Medicine and Health Sciences, University of Alcalá, 28801 Alcalá de Henares, Spain; 9 Immune System Diseases-Rheumatology and Internal Medicine Service, University Hospital Príncipe de Asturias, CIBEREHD, 28806 Alcalá de Henares, Spain

**Keywords:** pregnancy-induced CVD, chronic venous disease, proinflammatory cytokines, hypertensive vascular disorder, MeSH

## Abstract

Chronic venous disease (CVD) is a common vascular disorder characterized by increased venous hypertension and insufficient venous return from the lower limbs. Pregnancy is a high-risk situation for developing CVD. Approximately a third of the women will develop this condition during pregnancy, and similarly to arterial hypertensive disorders, previous evidence has described a plethora of alterations in placental structure and function in women with pregnancy-induced CVD. It is widely known that arterial-induced placenta dysfunction is accompanied by an important immune system alteration along with increased inflammatory markers, which may provide detrimental consequences for the women and their offspring. However, to our knowledge, there are still no data collected regarding cytokine profiling in women with pregnancy-induced CVD. Thus, the aim of the present work was to examine cytokine signatures in the serum of pregnant women (PW) with CVD and their newborns (NB). This study was conducted through a multiplex technique in 62 PW with pregnancy-induced CVD in comparison to 52 PW without CVD (HC) as well as their NB. Our results show significant alterations in a broad spectrum of inflammatory cytokines (IL-6, IL-12, TNF-α, IL-10, IL-13, IL-2, IL-7, IFN-γ, IL-4, IL-5, IL-21, IL-23, GM-CSF, chemokines (fractalkine), MIP-3α, and MIP-1β). Overall, we demonstrate that pregnancy-induced CVD is associated with a proinflammatory environment, therefore highlighting the potentially alarming consequences of this condition for maternal and fetal wellbeing.

## 1. Introduction

Chronic venous disease (CVD) is a common vascular disorder characterized by insufficient venous return from the lower extremities and an increase in venous pressure, known as venous hypertension [1,2]. Clinical manifestations of CVD can range from mild to severe, such as telangiectasia, venous ulceration, lipodermatosclerosis, and, most notably, varicose veins. Risk factors for developing CVD include advanced age, obesity, genetics, and female sex [3,4,5]. Pregnancy also appears to be a major risk factor for developing CVD. Indeed, it is estimated that approximately 40% of women will suffer from this condition during pregnancy, and this increased risk is even higher with the number of pregnancies [6,7]. During pregnancy, there are many changes in the hemodynamics of the cardiovascular system accompanied by hormonal and mechanical variations. Some of these changes include vasodilation, iliac vein compression, stasis, decreased venous flow velocity, and venous valve incompetence, hence leading to the development of CVD [8,9,10,11,12]. Previous studies have demonstrated the impact of CVD-induced venous hypertension on placental integrity. Specifically, evidence of cellular damage, hypoxia, increased calcification, oxidative stress, and increased vascularization was observed in the placentas of CVD patients [13,14,15,16]. These pathological manifestations are also observed in pre-eclampsia, an analogous condition also characterized by vascular hypertension [2,17,18]. However, the effects of CVD on maternal and fetal wellbeing have not been as widely studied as pre-eclampsia.

The pathogenesis of CVD in pregnancy remains poorly understood. Cytokines are central players in immune system modulation and also show modulatory effects on different tissues and organs [19]. Alterations in cytokine production and circulating levels have been involved in the pathogenesis of organ and systemic damage [20]. Different patterns of variation in cytokine serum levels have been to be associated with different inflammatory diseases [21]. They may provide either beneficial effects, collaborating with host defense, or they can be related to adverse outcomes under pathological conditions when they are dysregulated [19]. Pregnancy is, in part, inflammatory status, and a broad range of studies have demonstrated the relevance of analyzing cytokine signatures in both physiological and pathological conditions [22,23,24,25,26,27]. Conversely, elevated levels of inflammatory cytokines, such as TNF-α and IL-6, have been implicated in the pathogenesis of vascular hypertension during pregnancy [28,29]. Inflammation often mediates the deterioration of healthy tissue, and proinflammatory cytokines instigate apoptotic pathways responsible for many of the clinical presentations of CVD [30]. It has been shown that proinflammatory cytokines are significantly elevated in patients with CVD compared to healthy controls. On aggregate, these previous data on proinflammatory cytokine levels in both CVD and pregnancy-induced vascular hypertension suggest that proinflammatory cytokines play an important role in pregnancy-induced CVD. This study aims to identify the systemic effects of pregnancy-induced CVD by measuring proinflammatory cytokines in the peripheral blood of mothers as well as of their newborns.

## 2. Results

### 2.1. Women with CVD during Pregnancy Show an Increase in Different Proinflammatory Cytokines

**Figure 1:**
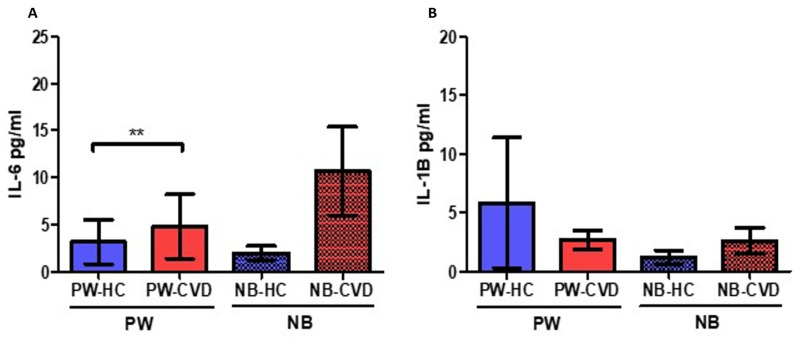
Histogram showing the significant increase in IL-6 in PW-CVD but not in their NB (**A**). IL-1B did not show any variation neither in the plasma of PW-CVD or NB-CVD (**B**). PW-HC = pregnant women without vascular pathology, PW-CVD = pregnant women with chronic venous disease during gestation, NB-HC = newborns of mothers without vascular pathology, NB-CVD = newborns of mothers with chronic venous disease during gestation. *p* < 0.01 (**).

Analysis of plasma samples has shown a significant increase in many proinflammatory cytokines. For instance, we observed a significant increase in IL-6 levels in the plasma of PW-CVD patients (PW-HC = 3.168 ± 4.897 pg/mL vs. PW-CVD = 4.791 ± 314.900 pg/mL, *p* ** = 0.029, Figure 1A). This trend of a significant increase is similarly observed in the plasma of NB-CVD without being statistically significant (NB-HC = 1.988 ± 1.555 pg/mL vs. NB-CVD = 10.685 ± 19.350 pg/mL, *p* = 0.3167, Figure 1A). In contrast, no significant differences were observed in the plasma levels of IL-1B in the study patients (PW-HC = 5861 ± 11,486 pg/mL vs. PW-CVD = 2.692 ± 3.598 pg/mL, *p* = 0.9302, NB-HC = 1.231 ± 1.220 pg/mL vs. NB-CVD = 2.625 ± 4.536 pg/mL, *p* = 0.8366, Figure 1B).

**Figure 2:**
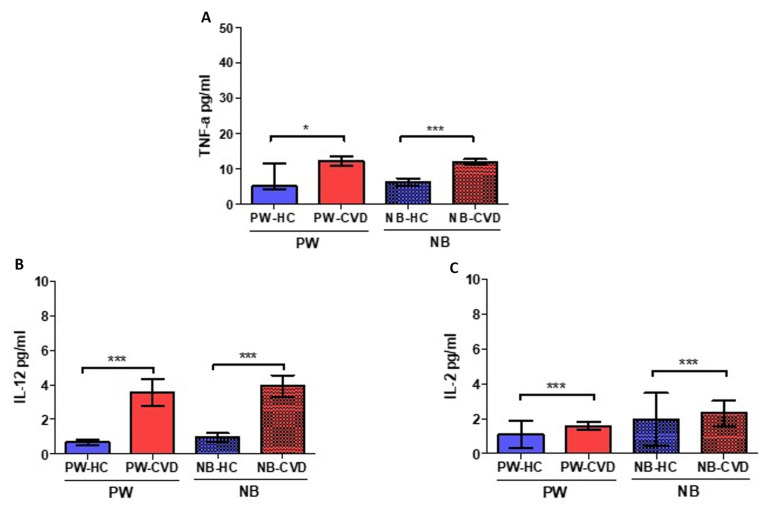
Histogram showing the significant increase in TNF-a (**A**), IL-12 (**B**), and IL-2 (**C**) in PW-CVD plasma and in NB-CVD. PW-HC = pregnant women without vascular pathology, PW-CVD = pregnant women with chronic venous disease during gestation, NB-HC = newborns of mothers without vascular pathology, NB-CVD = newborns of mothers with chronic venous disease during gestation. *p* < 0.05 (*), *p* < 0.001 (***).

Simultaneously, an increase in TNF-α was reported in pregnant women with CVD (PW-CVD) compared to PW-HC (PW-HC = 14.002 ± 23.096 pg/mL vs. PW-CVD = 12,295 ± 5477 pg/mL, *p* * = 0.0167, Figure 2A). Likewise, NB-CVD display a notable increase in this cytokine in comparison to NB-HC (NB-HC = 6225 ± 2360 pg/mL vs. NB-CVD = 12,076 ± 3079 pg/mL, *p* *** < 0.0001). Likewise, our results reported a significant increase in the proinflammatory cytokine IL-12 in pregnant women with CVD (PW-CVD) compared to PW-HC, as well as in NB-CVD (PW-HC = 0.698 ± 0.320 pg/mL vs. PW-CVD = 3.569 ± 1.617 pg/mL, *p* *** < 0.0001, NB-HC = 0.965 ± 0.469 pg/mL vs. NB-CVD = 3917 ± 1335 pg/mL, *p* *** < 0.0001, Figure 2B). Subsequently, a significant increase in the proinflammatory cytokine IL-2 was observed in PW-CVD with respect to PW-HC, as well as in NB-CVD (PW-HC = 1112 ± 3343 pg/mL vs. PW-CVD = 1583 ± 0.987 pg/mL, *p* *** < 0.0001, NB-HC = 1948 ± 6268 pg/mL vs. NB-CVD = 2309 ± 3108 pg/mL, *p* *** = 0.0002, Figure 2C).

**Figure 3:**
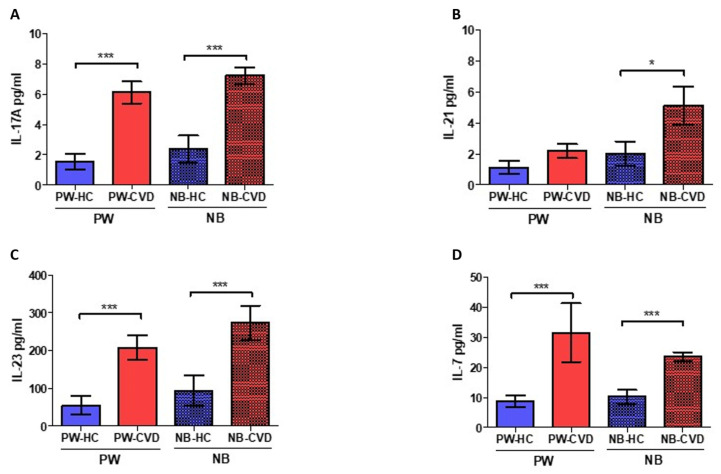
Histogram showing a significant increase in IL-17A (**A**), IL-23 (**C**), and IL-7 (**D**) in PW-CVD plasma and in NB-CVD. Likewise, an increase in IL-21 (**B**) in NB-CVD is also observed. PW-HC = pregnant women without vascular pathology, PW-CVD = pregnant women with chronic venous disease during gestation, NB-HC = newborns of mothers without vascular pathology, NB-CVD = newborns of mothers with chronic venous disease during gestation. *p* < 0.05 (*), *p* < 0.001 (***).

Similarly, IL-17A levels were significantly higher in PW-CVD compared to PW-HC, as well as in NB-CVD (PW-HC = 1555 ± 1055 pg/mL vs. PW-CVD = 6119 ± 3244 pg/mL, *p* *** < 0.0001, NB-HC = 2384 ± 1711 pg/mL vs. NB-CVD = 7245 ± 2381 pg/mL, *p* *** < 0.0001, Figure 3A). No significant differences were observed in plasma levels of IL-21 in PW-CVD with respect to PW-HC (PW-HC = 1142 ± 0.843 pg/mL vs. PW-CVD = 2197 ± 1991 pg/mL, *p* = 0.0871, Figure 3B). However, plasma IL-21 levels were significantly higher in NB-CVD compared to NB-HC (NB-HC = 2036 ± 1501 pg/mL vs. NB-CVD = 5.124 ± 5.428 pg/mL, *p* * = 0.0174, Figure 3B). Moreover, we found a significant increase in the proinflammatory cytokine IL-23 in pregnant women with CVD (PW-CVD) compared to PW-HC, as well as in NB-CVD (PW-HC = 54,831 ± 51,632 pg/mL vs. PW-CVD = 208.095 ± 144.753 pg/mL, *p* *** < 0.0001, NB-HC = 93.715 ± 78.808 pg/mL vs. NB-CVD = 273,872 ± 196,395 pg/mL, *p* *** = 0.0008, Figure 3C). Similarly, the levels of the proinflammatory cytokine IL-7 were significantly higher in PW-CVD, being similar in NB-CVD (PW-HC = 8778 ± 4162 pg/mL vs. PW-CVD = 31,549 ± 42,609 pg/mL, *p* *** < 0.0001, NB-HC = 10,281 ± 4534 pg/mL vs. NB-CVD = 23,503 ± 6550 pg/mL, *p* *** < 0.0001, Figure 3D). 

### 2.2. Women with CVD during Pregnancy Show a Decrease in Anti-Inflammatory Cytokines

**Figure 4:**
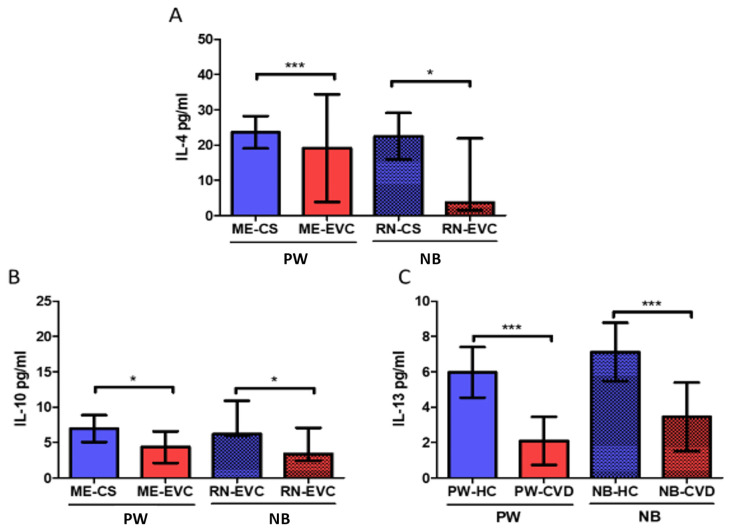
Histogram showing the significant decrease in the anti-inflammatory cytokines (**A**) IL-4, (**B**) IL-10, and (**C**) IL-13 in PW-CVD plasma and in NB-CVD. PW-HC = pregnant women without vascular pathology, PW-CVD = pregnant women with chronic venous disease during gestation, NB-HC = newborns of mothers without vascular pathology, NB-CVD = newborns of mothers with chronic venous disease during gestation. *p* < 0.05 (*), *p* < 0.001 (***).

Our results showed a significant decrease in IL-4 levels in PW with CVD (PW-CVD) with respect to PW-HC (PW-HC = 23,642 ± 19,978 pg/mL vs. PW-CVD= 19,149 ± 66,704 pg/mL, *p* *** < 0.0001, Figure 4A). In parallel, we have observed a decrease in IL-4 in the umbilical cord plasma of NB-CVD with respect to NB-HC (NB-HC = 22,469 ± 13,756 pg/mL vs. NB-CVD = 37,471 ± 88,639 pg/mL, *p* * = 0.0265, Figure 4A). Furthermore, our analyses have shown a significant decrease in the anti-inflammatory cytokine IL-10 in PW with CVD (PW-CVD) with respect to PW-HC (PW-CVD = 6958 ± 3949 pg/mL vs. PW-HC = 4.354 ± 4.596 pg/mL, *p* * = 0.0102, Figure 4B). In parallel, we have observed a decrease in IL-10 in the umbilical cord plasma of NB-CVD with respect to NB-HC (NB-CVD = 8611 ± 5201 pg/mL vs. NB-HC = 8.307 ± 16.487 pg/mL, *p* * = 0.0127, Figure 4B). Our analysis has reported a significant decrease in the anti-inflammatory cytokine IL-13 in PW-CVD with respect to to PW-HC (PW-CVD = 2094 ± 5902 pg/mL vs. PW-HC = 5973 ± 2975 pg/mL, *p* *** < 0.0001, Figure 4C). Simultaneously, we have observed a decrease in IL-13 in the umbilical cord plasma of NB-CVD with respect to NB-HC (NB-CVD = 3453 ± 7995 pg/mL vs. NB-HC = 7.121 ± 3.439 pg/mL, *p* *** = 0.0002, Figure 4C).

### 2.3. Women with CVD during Pregnancy Showed a Decrease in IFN-ɣ

**Figure 5:**
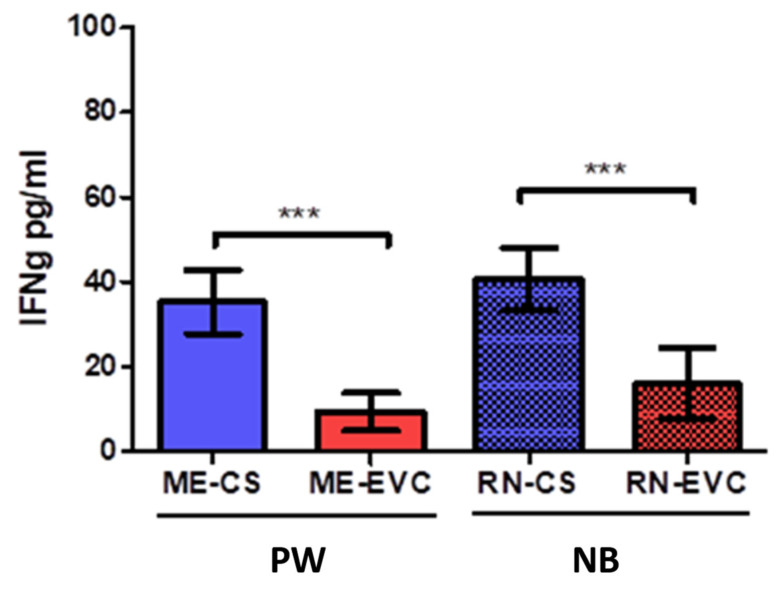
Histogram showing a significant decrease in IFN-ɣ in PW-CVD plasma and in NB-CVD. PW-CVD = pregnant women with chronic venous disease during gestation, NB-CVD = newborns of mothers with chronic venous disease during gestation. *p* < 0.001 (***).

A significant decrease in INF-ɣ levels was observed in pregnant women with CVD (PW-CVD) with respect to PW-HC (PW-HC = 35,221 ± 15,473 pg/mL vs. PW-CVD = 9390 ± 9130 pg/mL, *p* *** < 0.0001, Figure 5. In parallel, we have observed a decrease in INF-ɣ in the umbilical cord plasma of NB-CVD with respect to NB-HC (NB-HC = 40,815 ± 15,181 pg/mL vs. NB-CVD = 15,969 ± 16,285 pg/mL, *p* *** < 0.0001, Figure 5).

### 2.4. Women with CVD during Pregnancy Show an Increase in the Eosinopoietins GM-CSF and IL-5 

**Figure 6:**
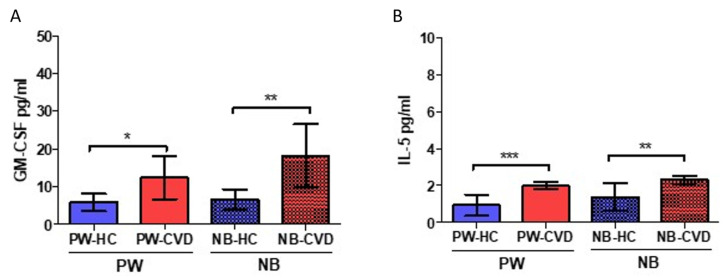
Histogram representing the significant increase in GM-CSF (**A**) and IL-5 (**B**) in plasma from PW-CVD and NB-CVD. PW-HC = pregnant women without vascular pathology; PW-CVD = pregnant women with chronic venous disease during pregnancy; NB-HC = newborn from mothers without vascular pathology; NB-CVD = newborn from mothers with CVD during pregnancy. *p* < 0.05 (*), *p* < 0.01 (**), *p* < 0.001 (***).

Analysis of plasma samples has shown a significant increase in GM-CSF in pregnant women with CVD (PW-CVD) compared to PW-HC (PW-HC = 5900 ± 3276 pg/mL vs. PW-CVD = 12,359 ± 10,980 pg/mL, *p* * = 0.0104, Figure 2). In parallel, we have observed an increase in GM-CSF in the umbilical cord plasma of NB-CVD with respect to NB-HC (NB-HC = 6567 ± 5234 pg/mL vs. NB-CVD = 18.108 ± 17.329 pg/mL, *p* ** = 0.0084, Figure 6A).

Similarly, IL-5 levels were significantly higher in PW-CVD compared to PW-HC, as well as in NB-CVD (PW-HC = 0.936 ± 1.196 pg/mL vs. PW-CVD = 1.987 ± 0.884 pg/mL, *p* *** = 0.0002, NB-HC = 1.387 ± 1.447 pg/mL vs. NB-CVD = 2.316 ± 0.957 pg/mL, *p* *** = 0.0019, Figure 6B).

### 2.5. Women with CVD during Pregnancy Show a Significant Increase in Plasmatic Chemokines

**Figure 7:**
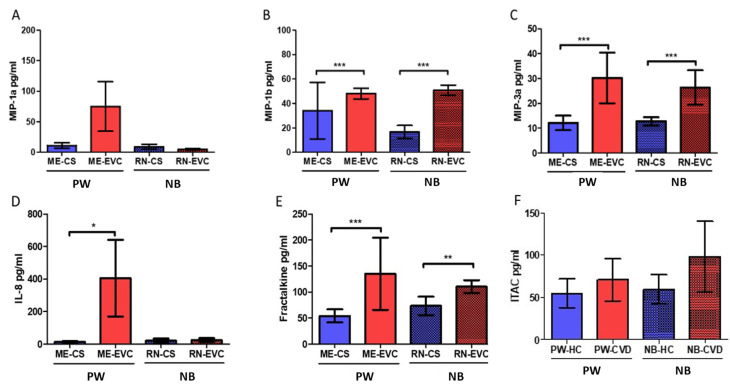
Histogram representing a significantly increased level of plasmatic chemokines (**A**) MIP-1a, (**B**) MIP-1b, (**C**) MIP-3a, (**D**) IL-8, (**E**) Fractalkine, and (**F**) ITAC in PW-CVD and in NB-CVD. PW-CVD = pregnant women with chronic venous disease during pregnancy; NB-HC = newborn from mothers without vascular pathology; NB-CVD = newborn from mothers with CVD during pregnancy. *p* < 0.05 (*), *p* < 0.01 (**), *p* < 0.001 (***).

The study of plasma samples did not show significant differences in MIP-1a levels in PW-CVD compared to PW-HC, just as in NB (PW-HC = 11.153 ± 19.986 pg/mL vs. PW-CVD = 75.099 ± 176.796 pg/mL, *p* = 0.8674, NB-HC = 9.165 ± 7.764 pg/mL vs. NB-CVD = 5.077 ± 3.881 pg/mL, *p* = 0.1322, Figure 7A). On the contrary, a significant increase in MIP-1b was observed in PW-CVD compared to PW-HC, just as in NB (PW-HC = 34.131 ± 47.936 pg/mL vs. PW-CVD = 48.097 ± 19.065 pg/mL, *p* = *** 0.007, NB-HC = 16.585 ± 11.144 pg/mL vs. NB-CVD = 550.822 ± 17.412 pg/mL, *p* *** < 0.0001, Figure 7B). In this line, a significant increase in MIP-3a was evinced in PW-CVD compared to PW-HC, just as in NB (PW-HC = 12.096 ± 6.086 pg/mL vs. PW-CVD = 30.241 ± 21.189 pg/mL, *p* = *** 0.0003, NB-HC = 12.759 ± 3.407 pg/mL vs. NB-CVD = 26.338 ± 13.532 pg/mL, *p* *** < 0.0001, Figure 7C).

Our results have demonstrated a significant increase in IL-8 plasmatic levels in PW-CVD; however, an upward trend was only observed in NB-CVD (PW-HC = 14.050 ± 19.501 pg/mL vs. PW-CVD = 405.486 ± 915.893 pg/mL, *p* = * 0.0209, NB-HC = 20.878 ± 25.607 pg/mL vs. NB-CVD = 25.127 ± 48.059 pg/mL, *p* = 0.3581, Figure 7D). Moreover, a significant increase in fractalkine was observed in PW-CVD compared to PW-HC (PW-HC = 54.148 ± 26.064 pg/mL vs. PW-CVD = 135.082 ± 143.891 pg/mL, *p* *** < 0.0001, Figure 7E). Moreover, significant increased levels were observed in NB-CVD’s umbilical cord compared to NB-HC (NB-HC = 73.367 ± 34.607 pg/mL vs. NB-CVD = 110.285 ± 26.028 pg/mL, *p* ** = 0.0022, Figure 7E). 

Finally, the study of plasma samples has not shown significant differences either in ITAC levels in PW-CVD with respect to PW-HC or NB-CVD’s umbilical cord compared to NB-HC (PW-HC = 54,823 ± 35,371 pg/mL vs. PW-CVD = 70,630 ± 51,407 pg/mL, *p* = 0.3209, NB-HC = 59,398 ± 35,982 pg/mL vs. NB-CVD = 97.948 ± 814.592 pg/mL, *p* = 0.2540, Figure 7F).

## 3. Discussion

In the present work, we have demonstrated that CVD leads to an altered cytokine signature in the PW and their NB in comparison to those without this condition. More detailly, we have observed an increased serum level of several proinflammatory cytokines but reduced levels of anti-inflammatory cytokines and INF-ɣ. Simultaneously, we have observed raised serum chemokines and GCSF, measurable in the PW with CVD and their NB.

CVD is a multifactorial disease with complex pathophysiological mechanisms involved, associated with an important inflammatory response [31]. CVD involves a powerful alteration in the immune inflammatory system, with a significant increase in plasmatic innate and adaptive cytokines. In fact, it has been demonstrated that CVD itself causes noteworthy changes in cytokine production by the immune cells, hence inducing proinflammatory profiling [32]. CVD has also been associated with placental, umbilical cord, and systemic oxidative stress [14,33]. Moreover, an altered local detection of some cytokines has also been observed in the placenta of women with CVD, which is closely related to abnormal cell and molecular behavior [34,35,36]. This inflammatory, hypoxic, and also oxidative environment could be part of fetal programming, as some previous studies suggest [37,38,39]. In this sense, we show the possible role of a group of cytokines in PW undergoing CVD that could simultaneously affect NB, showing a proinflammatory state. To our knowledge, our study is the first to evidence a unique cytokine signature in this group of patients, therefore showing that CVD may be a deteriorating condition with important consequences for both PW and NB. 

Cytokines can be classified according to different criteria such as molecular composition, interaction receptor, main cell producers, and targets [40]. However, from a pathogenic point of view, cytokines are defined by their effect on the immunoinflammatory response as proinflammatory and anti-inflammatory. They are produced by leukocytes and other cells, being essential to orchestrate immune cells growth, differentiation, and activation [41]. Moreover, many of these cytokines have provided their clinical relevance in a wide variety of conditions, including during normal pregnancy or its complications [26,29]. This is mainly due to the fact that many of these cytokines can cross the placental barrier, although it is in dispute to what extent this fact occurs [42]. In our study, we found significant alterations in diverse interleukins, including IL-6, IL-2, IL-12, IL-7, IL-21, IL-23, IL-10, IL-13, IL-4, and IL-5. 

Our data clearly show a marked increase in serum levels of proinflammatory cytokines in PW-CVD. Unfortunately, this maternal immunoinflammatory disturbance is also found in NB-CVD. We have found increased IL-6 and TNF-α serum levels in both populations. In agreement with this fact, we also observed increased IL-6 levels in the placenta of women with CVD [34]. IL-6 is mostly a proinflammatory but also anti-inflammatory cytokine with pleiotropic effects in the organism [43]. For instance, IL-6 participates in B-cell differentiation and stimulation of acute phase proteins [41]. Increased maternal IL-6 levels have been related to the development and severity of different pregnancy-associated complications such as pre-eclampsia or chorioamnionitis [44,45]. IL-6 with TNF-α exert synergistic proinflammatory effects [46]. It seems that high levels of both cytokines promote trophoblasts cell death, hence impairing placental function [47]. In addition, high levels of IL-6 in the umbilical cord have been associated with the requirement of oxygen at 36 weeks of post-menstrual age in small for gestational age newborns [48]. Furthermore, increased maternal serum levels of IL-6 and TNF-α have been associated with hypertensive disorders during pregnancy [49,50,51]. In this line, our study might indicate an important correlation between CVD with high levels of IL-6 and TNF-α. Because of that, TNF-α has been proposed as a potential target for preventing placental and fetal complications of pregnancy [52]. Further studies could be designed to evaluate therapeutical approaches of TNF-α inhibitors in pregnant women with CVD as well as to avoid possible fetus or newborn complications. Moreover, we cannot disregard the fact that IL-6 has also been described as an elevated inflammatory mediator during labor onset [50], but this condition was shared by both groups of women. 

In contrast with the increased serum levels of IL-6 with TNF-α, our results show normal concentrations of IL-1β. Different patterns of alterations in the levels of these cytokines have been observed in inflammatory diseases, as well as different clinical responses to specific anti-cytokine treatments [53]. These findings suggest that the cellular mechanisms involved in the pathogenesis of the proinflammatory status of PW-CVD are specific.

Associated with the systemic proinflammatory environment observed in PW-CVD, we have found a marked disbalance of the circulating cytokines secreted by the different Th subsets. An increase in Th1 cytokines has been observed in these women and their NB. IL-2, IL-12, and TNF-α are critical cytokines involved in Th1 responses, while IL-4 and IL-10 inhibit this polarization [54]. IL-2, also called T-cell growth factor, is a central cytokine involved in the proliferation and differentiation of both adaptative and innate immune cells [55]. IL-2 is produced by polarized Th1 cells, and it has central effects on the activation of B cells, monocytes, natural killers (NKs), innate lymphoid cells (ILCs), as well as modulating effector T cells and T reg activity [56]. However, it is hypothesized that IL-2 proinflammatory/anti-inflammatory effects might be determined by the amount and kinetics of IL-2. Thus, a high but transient level of IL-2 appears to be associated with effector cell development, while low-grade IL-2 presence could be related to T reg induction [57]. T reg populations are essential for gestational success, and a correct IL-2—STAT5 signaling with adequate levels of T reg has been associated with the prevention of autoimmunity and human recurrent abortions [58]. Oppositely, increased levels of IL-2 have been related to higher NK cytotoxicity, which has been proposed as a risk factor for human recurrent abortions [59]. Increased IL-2 levels described in PW and NB related to CVD may indicate a likely imbalance of Th1/T regs and NK cytotoxicity, therefore supporting a proinflammatory status affecting both individuals. Moreover, we have also reported increased levels of further Th1 cytokines, including the proper TNF-α and IL-12, along with a reduction in IL-4 and IL-10. IL-12 is a crucial cytokine involved in IFN-γ production [60], also related to pathogenic Th1 differentiation [61]. Simultaneously, IL-12 is also associated with an imbalance in Th1/Th2 cells, which has been associated with pregnancy complications such as recurrent spontaneous abortion, obstetric complications, and poor pregnancy outcomes [62]. Despite the elevated IL-12 levels found, we report a significant IFN-γ decrease in both PW with CVD and NB. The role of IFN-γ in pregnancy has already been well-described and substantial alterations of this cytokine appear to be related to different pregnancy complications such as preterm labor [63]. In this line, Scott et al. [64] also reported high levels of IL-12 without IFN-γ induction by immune cells extracted from cord blood. More recently, a reduction in IFN-γ levels was observed in PW with pre-eclampsia [65]. It is probable that IFN-γ diminishment could be associated with pathological conditions such as CVD, although further works should clarify the mechanisms involved in its dysregulation.

IL-4, IL-10, and IL-13 are three anti-inflammatory cytokines significantly decreased in our study in both PW and NB. IL-10 was first discovered as a product secreted by Th2 cells, although this cytokine is secreted by many types of immune cells, being capable of reducing proinflammatory cytokines release and Th1 responses [66,67]. It causes inhibition of IL-2 and interferon gamma [41]. One of the most important roles of IL-10 is to provide contrary effects to TNF-α. Thus, an adequate balance between IL-10 and TNF-α is crucial during pregnancy, and reductions in IL-10 levels with augmented TNF-α might be related to pathological inflammation during this period [68]. In addition, deficiencies in IL-4 and IL-10 cytokines have been associated with a plethora of pregnancy-related disorders, including infertility, spontaneous miscarriage, preterm birth, fetal growth restriction, pre-eclampsia, gestational hypertension [69], and as we have just demonstrated with CVD. IL-4 is synthesized by CD4+T cells, and it is a major inductor of Th2 differentiation while inhibiting Th1 phenotyping, acting co-ordinately with IL-13 in the alternative macrophage polarization (M2 responses), among other effects [70]. Animal models show that the absence of IL-4 is sufficient to induce pregnancy hypertension accompanied by excessive inflammation in IL-4-deficient mice [71]. Low levels of IL-4 and IL-10 have been described in pregnancies with severe pre-eclampsia [72]. Similarly, low maternal levels of IL-4 and IL-13 have been positively correlated with an increased risk of NB for developing overweight during childhood [73]. Therefore, our results might indicate the pathological role of IL-4, IL-10, and IL-13 reduction due to CVD, furthermore promoting a proinflammatory status and a Th1/Th2 imbalance, which has also been implicated with preterm labor [74]. 

Additionally, we have observed increased Th17 cytokine levels in PW with CVD and their NB. Accordingly, elevated levels of circulating IL-23 and IL-17A are found in PW-CVD and NB-CVD. IL-23 is a member of the IL-12 family, and it is key to inducing the Th17 pathogenic phenotype through the stabilization of IL-17 (Also known as IL-17A) [60]. Our results show increased IL-17A and IL-23 levels, denoting an abnormal Th17 polarization associated with CVD in PW and NB. IL-17A dysregulation is associated with the development and progression of different inflammatory diseases [75]. A study conducted Eghbal-Fard et al. [76] in 50 women with pre-eclampsia also reported the contribution of higher serum levels of IL-17A and IL-23 in the pathogenesis of the disease, with impaired Th17/Treg ratio. Conversely, other studies only detected significant differences in IL-17 but not in IL-23 [77,78]. In this line, we show that CVD is responsible for the induction of both IL-17 and IL-23 production, which may be implicated in the proper pathogenesis of the disease. In the same line, we report a significant increase in IL-7 in both maternal and fetal serum. IL-7 is crucial for B-cell proliferation, T-cell development, and homeostasis [79]. Additionally, it has been associated with pregnancy complications such as recurrent pregnancy losses due to its ability to induce aberrant Th17 responses and reductions in Treg cells in animal models [80]. In addition, it has been proposed that IL-7 crosses the placental barrier and triggers IL-17R, and could affect fetal neurons producing cortical and behavioral abnormalities [24]. Furthermore, we reported an increased IL-21 in the cord blood obtained from NB. IL-21 is another cytokine produced by T cells and NKT cells, inducing Th17 phenotyping while stimulating NKT, NK, and T cytotoxic subsets proliferation and cytotoxicity [81]. However, in immune cells derived from cord blood, IL-21 seems to stimulate the expression of immunosuppressive IL-10 to diminish Th1 responses [82]. It is probable that increased levels of IL-21 in the NB could emerge as a protective mechanism to diminish the global proinflammatory status. 

IL-5 causes B-cell growth factor and differentiation and IgA selection. IL-5, together with granulocyte-macrophage colony-stimulating factor (GM-CSF), plays a key role in eosinophilic function and development, being frequently designed as “eosinopoietins” [83]. Previous research has established the synergic action of GM-CSF and IL-5 on eosinophil activation under inflammatory diseases [84]. Although, classically, the eosinophils were associated with anti-parasite responses, nowadays, it is widely accepted their importance in maintaining tissue homeostasis [85]. Furthermore, eosinophils are involved in the secretion of many immunomodulatory cytokines, integrating different signals and directing inflammatory responses [86]. An altered eosinophilic activity might be a clinical risk of note during mild to late gestation of preterm labor related to type I hypersensitivity reaction [87]. Recently, Lebold et al. [88] have demonstrated that intra-utero exposition to IL-5 results in fetal eosinophilia and as a developmental origin of airway hyperreactivity in the adult offspring. Regarding GM-CSF, this cytokine is produced by different cells from the innate and adaptative immune system with major effects in bone marrow, where stem cells are provoked to mature not only into eosinophils but also into monocytes and macrophages [89]. GM-CSF is importantly produced both by Th17 and Th1 cells after IL-23 and IL-12, respectively [90,91]. In addition, the IL-7 axis is involved in GM-CSF production by Th subsets that could lead to autoimmune diseases such as type 1 diabetes mellitus [92]. Besides its role in cell growth, it may act as a proinflammatory cytokine in infections and activates the following pathways: JAK/STAT, PI3K, MAPK, and NFκB [93]. This factor has an important role in fertility and in embryo implantation and is crucial for placental development [94]. In fact, reduced levels of this cytokine during pregnancy were related to recurrent miscarriages [95], placental dysfunction, and abnormal fetal growth [96]. Conversely, Huang et al. [97] described the crucial role of aberrant GM-CSF expression in the pathogenesis of pre-eclampsia, acting as a powerful inductor of inflammatory cells. Increased levels of IL-5 and GM-CSF may indicate an abnormal activation of eosinophils in pregnancy-associated CVD. Future studies should be conducted to unravel the possible role of eosinophils in the pathogenesis of the disease and its consequences in newborns. 

Chemotactic cytokines are produced by mast cells and stimulate the migration of several cells, mostly WBCs, not only being involved in all kinds of immune responses but also in many other biological processes such as angiogenesis, embryonic development, phagocytosis, survival, and apoptosis [98]. By following gradients of several kinds of chemokines, cells are usually guided to the site of interest in homeostasis and inflammation and linking innate and adaptative responses [99]. In this study, there were four chemokines found from two different subfamilies, CC and CXC (classification is based upon cysteine residues position): CCL4, CCL20, IL-8, and CX3CL1, all of them with significance in the PW and the NB. CXC chemokines convey chemotactic activity for neutrophils and CC for monocytes and Th subsets, although there are exceptions [100]. Chemokines are critical regulators for trophoblasts invasion. The rising evidence alleges that chemokines are considered regulatory molecules that, due to their selective trafficking of immune cells, settle a normal or a pathological placental status and delivery [101]. The expression of chemokines in the endometrium orchestrates the appropriate infiltration of immune cells and invasion of trophoblasts in the maternal vasculature. It is known that trophoblast cells express countless membrane receptors for these chemokines as well, contributing to fetal immunity besides placental development. 

IL-8 (CXCL8) is released by NK cells implying the migration of trophoblast cells for endovascular invasion and maternal vascular remodeling [102]. Elevated levels of cord blood IL-8 have been associated with pre-eclampsia [103] and moderate-severe bronchopulmonary dysplasia in NBs [48]. The chemokine network at the fetal-maternal interface also looks decisive in the future adult’s health. Our results denote a decrease in the inhibitors of IL-8, which are anti-inflammatory cytokines IL-4 and IL-13. When blocking IL-8, and hence neutrophil migration, by these two, the polarization of Th subsets tends to Th2 type [104]. As in this case, there is no impedance for IL-8 activity together with other chemokines and interleukins, and Th2 response is decreased. We also found increased TNF-α, which upregulates IL-8 [105], agreeing with the high IL-8 obtained. At the same time, at normal term, choriodecidua and amnion also produce IL-8 [50], boosting the activity of MMPs and other compounds; meanwhile, IL-6 and TNF-α also stimulate these components leading to collagenolysis [101]. In previous studies, we found increased levels of MMP-9 and COL-III, affecting the structure of the placentas of women with venous insufficiency during pregnancy [106]. Then, if certain chemokines may upregulate the expression of collagenolytic components, CVD may increase these even more. 

Furthermore, we found significant plasma levels of fractalkine (CX3CL1) and CCL4 (MIP-1β). Some studies have found an association between fractalkine and later pre-eclampsia, concretely decidual cell secreted CX3CL1 but not circulating [107]. Other trials have denoted that pregnant women with pre-eclampsia present an overexpression of fractalkine, coinciding with poor pregnancy outcomes [108]. Recently, elevated levels of fractalkine in maternal serum in pre-eclampsia have been described [109]. Moreover, CX3CL1 chemoattractant and adhesive properties breeding inflammation and angiogenesis processes are especially upregulated by inflammatory conditions such as diabetic placenta. The evidence also demonstrates a robust upregulation by hypoxia conditions [110]; hence, we could see CVD women also have overexpression of fractalkine compared to the control group, which could be promoted by hypoxia pathways such as HIF-1α, as we previously found in placenta from women with venous insufficiency [18]. CCL4 has been observed with enhanced expression related to implantation competence, serving as a predictor of pregnancy labor [111]. Increased detection of serum CCL4 has been associated with the presence of active infections during pregnancy [112]. Trophoblast migration also reacts to CCL4 and CX3CL1, being also key for maternal-fetal communication [113]. 

Finally, CCL20 (MIP-3α) was significantly increased in PW affected by CVD and their NB as well. CCL20 is known to be chemotactic and antimicrobial [114], and evidence says that it is a Th-17 response associated with chemokine, inducing inflammation [115]. The presence of this cytokine within amniotic fluid has been associated with microbial invasion and amniotic inflammation in preterm labors [111]. In the absence of infection, the bioavailability of CCL20 in amniotic fluid was associated with the partum process. However, it remains elusive if maternal serum concentrations of CCL20 might be indicative of intra-amniotic infection or inflammation [116]. 

Taken together, our results demonstrate a severe disturbance of cytokines and chemokines in PW with CVD and their NB. The interactions and possible implications of the abnormal pool of the cytokines mentioned before and the NB remains to be explored. In Table 1, the main findings and discussion about the different cytokines detected in our study are summarized. 

## 4. Materials and Methods

### 4.1. Experimental Design

An observational, analytical, and prospective cohort study was conducted on 114 pregnant women (PW) and their newborns (NB). A total of 62 plasma samples from PW diagnosed with CVD during pregnancy and their NB were obtained with a median age of 33 years (22–40) and a median gestational age of 40.5 weeks (39–41.5). Similarly, 52 plasma samples from PW and NB without CVD were also studied during pregnancy, with a median age of 34 years (27–41) and a median gestational age of 41 weeks (39–42).

Exclusion criteria were defined by women with endocrine diseases such as diabetes mellitus; high blood pressure (HBP); body mass index (BMI) > 25 kg/m^2^; unhealthy habits; active infectious diseases; autoimmune diseases; venous malformations; renal insufficiency; heart failure; pulmonary insufficiency; pre-eclampsia and/or hemolysis, elevated liver enzymes and low platelet syndrome (HELLP); uterine growth restriction of unknown cause; pathological lesions such as placental infarcts, avascular villi, late maturation and chronic inflammation affecting the chorionic villi; the appearance of any of these exclusion criteria described at any time before delivery or prior evidence of CVD.

All the participants were women who had visited their obstetrician at week 32 of gestation (time of blood sample collection). Once the informed consent was signed, her medical history was obtained, and a general physical exploration along with laboratory measurements was performed. An Echo-Doppler (portable M-Turbo Echo-Doppler; SonoSite, Inc., Washington, DC, USA) examination of the lower extremity was performed at 7.5 MHz while the women were in the orthostatic position, and the leg was examined by external rotation with support on the contralateral leg. The study included the greater saphenous axis from the inguinal region to the ankle and the femoral veins. Classification of CVD in participating PW was based on CEAP (Clinical-Etiological-Anatomical-Pathophysiological) [14]. All participants had CEAP scores ≥ 1 (C1 = 59.67% (*n* = 37), C2 = 33.87% (*n* = 21), C3 = 6.45% (*n* = 4)).

The gestational period of the studied participants was routinely monitored and followed at the Hospital Central de la Defensa Gómez Ulla-UAH (Madrid, Spain), and plasma samples were obtained from the umbilical cord vein at the time of delivery.

The study was carried out in accordance with the basic ethical principles of autonomy, beneficence, non-maleficence, and distributive justice, and its development followed the statements of Good Clinical Practice, the principles contained in the most recent Declaration of Helsinki (2013), and the Convention of Oviedo (1997). The data and information collected complied with current legislation on data protection (Organic Law 3/2018 of December 5, Protection of Personal Data and Guarantee of Digital Rights and Regulation (EU) 2016/679). The project was approved by the Clinical Research Ethics Committee of the Gómez Ulla Military Hospital (37/17).

### 4.2. Determination of Inflammatory Status

Plasma levels of ITAC, IL-10, granulocyte-macrophage colony-stimulating factor (GM-CSF), fractalkine, IFN-γ, MIP-3α, IL-12p70, IL-1β, IL-2, IL-5, IL-13, IL-21, IL-17A, IL-4, IL-23, IL-6, IL-7, IL-8, MIP-1α, MIP-1β, and TNFα were determined. With this aim, we used an aliquot of serum samples that had previously been obtained from peripheral blood in a dry tube by centrifugation at 2000 rpm for 20 min and kept at −80 °C until the moment of quantification. 

This study was carried out using the *Luminex* technique with a high sensitivity kit (Milliplex MAP kit, HSTCMAG-28SK-21) from the Merck laboratory (Darmstadt, Germany). For the study of cytokines, microspheres were used, each one encoded with a percentage of red and infrared depending on the element to be studied. These were incubated for 16–18 h with the antigen for binding to the capture antibody of each microsphere in 96-well plates. After incubation, the biotilinated detection antibody for every cytokine was added. Lastly, a streptavidin-phycoerythrin complex (Strep-PE) was employed, which bound the detection antibody. The plate was read on the MAGpix equipment (Merk). Using the standard curve, the Merck analysis program (Analyst) calculated the concentration of each cytokine of interest using the mean fluorescence intensity (MFI). Detection limits were established for all cytokines analyzed according to the protocol. 

### 4.3. Statistical Analysis 

For the statistical analysis, the GraphPad Prism^®^ 9.0 program (San Diego, CA, USA) was used, and the Mann–Whitney U test was applied. Data are expressed as the mean with SD. The significant results were established at *p* < 0.05 (*), *p* < 0.01 (**), and *p* < 0.001 (***).

## 5. Conclusions

**Figure 8:**
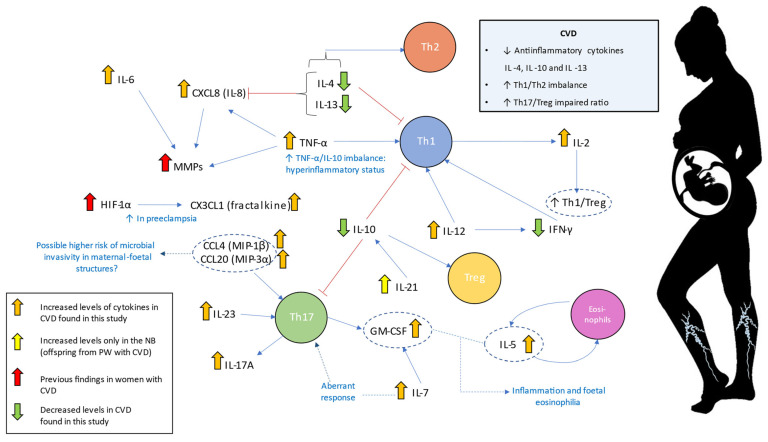
Summary of cytokines network studied in PW with CVD and their NB. The picture describes significant impaired levels of cytokines and conclusions from the study, being the Th1/Th2 imbalance a notable hallmark, besides the Th17/Treg impaired ratio. The complex network of signals could be determinant for the future child. A low level of anti-inflammatory cytokines is also associated with pre-eclampsia, gestational hypertension, spontaneous miscarriage, infertility, fetal growth restriction, and preterm birth. Low levels of IL-4 and IL-13 have been related to overweight in 1–2-year-old NB and could have their echo in health for adulthood. PW = pregnant woman; CVD = chronic venous disease; NB = newborn.

Overall, our study is the first to demonstrate a proinflammatory cytokine profiling in both PW and NB associated with pregnancy-induced CVD. This could have important consequences in the maternal and fetal environment, thereby affecting different immune populations from the innate and adaptative systems. Assuming the choriodecidual interface (where mother and fetal tissues are in contact) is a complex network of signals where each component (cells, cytokines, and many molecules) is a critical regulator, it is undeniable that the formed environment could be a determinant for the future child with echo in adulthood (as summarized in Figure 8). Future research could be approached to evaluate the impact of the inflammatory environment associated with CVD in women affected by this condition and their offspring.

## Figures and Tables

**Table 1:** Cytokines found in multiplex analysis. Results from PW with CVD and their NB, description, and possible implications. ↑ (increased), ↓ (decreased), - (no change), *p* < 0.05 (*), *p* < 0.01 (**), and *p* < 0.001 (***).

Significantly Altered Cytokines						
Cytokines	Original Designation	Abbreviatures	Targets and Functions	Pregnancy-Induced CVD	Previous Studies and Possible Implications	References
**Interleukins**	Interleukin-6	IL-6	A major proinflammatory cytokine. Synergic effects with TNF-α. B-cell differentiation and stimulation of acute phase proteins.	PW: ↑ ** NB: -	Increased maternal IL-6 levels have been related to the development and severity of different pregnancy-associated complications. High levels of IL-6 in the umbilical cord have been associated with the requirement of oxygen at 36 weeks of post-menstrual age in small for gestational age newborns.	[44,45,48]
	Interleukin-12	IL-12	Involved in pathogenic Th1 responses and IFN-γ production. It causes inhibition of IL-2 and interferon gamma.	PW: ↑ *** NB: ↑ ***	High IL-12 and low IFN-γ were observed in mononuclear cord blood cells. Th2-type response has been associated with pregnancy complications such as recurrent spontaneous abortion, obstetric complications, and poor pregnancy outcomes.	[41,62,64]
	Interleukin-10	IL-10	Anti-inflammatory cytokine Diminish Th1 responses and induce T reg activity.	PW: ↓ * NB: ↓ *	IL-10 and IL-4 reduction is associated with a plethora of pregnancy-related disorders, including infertility, spontaneous abortion, preterm birth, fetal growth restriction, pre-eclampsia, gestational hypertension	[68,69]
	Interleukin-13	IL-13	Anti-inflammatory effects acting synergically with IL-4 to promote Th2 responses	PW: ↓ *** NB: ↓ ***	Maternal levels of IL-4 and IL-13 were directly correlated with a decreased risk of NB for developing overweight in 1–2 years old	[73]
	Interleukin-2	IL-2	Pleiotropic effects on multiple immune populations. At high levels, it induces Th1 responses	PW: ↑ *** NB: ↑ ***	IL-2 dysregulation may negatively affect Treg expansion during pregnancy. Increased levels of IL-2 have been related to higher NK cytotoxicity, which has been proposed as a risk factor for human recurrent abortions.	[59,79]
	Interleukin-7	IL-7	Involved in T-cell development and homeostasis. B-cell proliferation.	PW: ↑ *** NB: ↑ ***	During pregnancy, IL-7 promotes an aberrant Th17 response with Treg reductions. Also, IL-7 could affect fetal neurons producing cortical and behavioral abnormalities.	[80]
	Interleukin-4	IL-4	Anti-inflammatory effects. IL-4 is a central inductor of Th2 responses and Th1 inhibition	PW: ↓ *** NB: ↓ *	IL-10 and IL-4 reduction are associated with a plethora of pregnancy-related disorders, including infertility, spontaneous miscarriage, preterm birth, fetal growth restriction, pre-eclampsia, gestational hypertension. Low maternal levels of IL-4 have been positively correlated with an increased risk of NB for developing overweight during childhood.	[69,73]
	Interleukin-5	IL-5	Together with GM-CSF and IL-3, they are “eosinopoietins” because of their ability to induce eosinophils proliferation and activation	PW: ↑ *** NB: ↑ **	An altered eosinophilic activity might be a clinical risk of preterm labor In utero exposition to IL-5 result in fetal eosinophilia and is a developmental origin of airway hyperreactivity	[87,88]
	Interleukin-17A	IL-17A	Along with IL-23, it mediates Th17 responses. Involved in the development of many inflammatory diseases	PW: ↑ *** NB: ↑ ***	Studies in women with pre-eclampsia show increased IL-17A levels alone or in combination with IL-23	[76,77,78]
	Interleukin-21	IL-21	Inductor of Th17 responses	PW: - NB: ↑ *	In cord blood cells, it may induce IL-10 production	[82]
	Interleukin-23	IL-23	Along with IL-17A, it mediates Th17 responses.	PW: ↑ *** NB: ↑ ***	Studies in women with pre-eclampsia show increased IL-17A in combination with IL-23	[76]
**Tumor necrosis factor**	Tumor necrosis factor-α	TNF-α	Proinflammatory cytokine that coordinates Th1 responses	PW: ↑ * NB: ↑ ***	High levels of TNF-α alone or with increased IL-6 and low IL-10 are related to pregnancy hypertensive disorders and other complications.	[24,47,51,52]
**Interferons**	Type II interferon gamma	IFN-γ	Proinflammatory cytokine that coordinates Th1 responses	PW: ↓ *** NB: ↓ ***	Low IFN-γ levels were detected in women with pre-eclampsia and blood cord despite high IL-12 levels.	[64,65]
**Colony-stimulating factors**	Granulocyte-macrophage colony-stimulating factor or colony-stimulating factor 2	GM-CSF (CSF-2)	Participates in Th1 and Th17 responses Together with IL-5 and IL-3, they are “eosinopoietins” because of their ability to induce eosinophils proliferation and activation	PW: ↑ * NB: ↑ **	Reduced levels of this cytokine were related to recurrent miscarriage, placental dysfunction, and abnormal fetal growth. Increased levels of this cytokine might be implicated in the pathogenesis of pre-eclampsia. An altered eosinophilic activity might be a clinical risk of preterm labor	[62,87,96,97]
**Chemokines**	Fractalkine or chemokine (C-X3-C motif) ligand 1	CX_3_CL1	Chemoatractive properties. Upregulated by hypoxia	PW: ↑ *** NB: ↑ **	Overexpression of this cytokine is related to poor pregnancy outcomes such as pre-eclampsia and gestational diabetes	[108,110]
	Chemokine (CXC motif) ligand-8 or Interleukin-8	CXCL8 (IL-8)	Neutrophils recruitment. Involved in Th1 responses and inhibited by Th2 cytokines (IL-4 and IL-13)	PW: ↑ NB: ↑	Some studies have found a positive correlation between maternal IL-8 levels and the risk of mental disorders in adulthood offspring. IL-8 induces matrix remodeling in placental tissue. Elevated levels of cord blood IL-8 have been associated with pre-eclampsia and moderate-severe bronchopulmonary dysplasia in newborns	[48,101,103]
	Macrophage inflammatory protein-1β or Chemokine (C-C motif) ligand 4	MIP-1β (CCL4)	Chemoattractive molecule of T lymphocites, dendritic cells, monocytes, and NKs; HIV correceptor	PW: ↑ *** NB: ↑ ***	Increased levels of this molecule appear to be indicative of active infections during pregnancy. Together with fractalkine, it is a central component in maternal-fetal dialogue	[112,113]
	Macrophage inflammatory protein-3α or chemokine (C-C motif) ligand 20	MIP-3α (CCL20)	Chemotactic and antimicrobial activity; associated with Th17 polarization and inflammation	PW: ↑ *** NB: ↑ ***	The presence of this cytokine in the amniotic fluid is a marker of infection or inflammation affecting the amniotic cavity. It remains to be elucidated is correlation with serum levels	[111,116]

## Data Availability

The data used to support the findings of the present study are available from the corresponding author upon request.
